# Secondary malignant giant cell tumor of bone due to malignant transformation 40 years after surgery without radiation therapy, presenting as fever of unknown origin: a case report

**DOI:** 10.1186/s13256-016-0833-7

**Published:** 2016-03-08

**Authors:** Hisataka Takesako, Eiji Osaka, Yukihiro Yoshida, Masahiko Sugitani, Yasuaki Tokuhashi

**Affiliations:** 1 Department of Orthopaedic Surgery, Nihon University School of Medicine, 30-1 Oyaguchikami-cho, Itabashi-ku, Tokyo, 173-8610 Japan; 2 Department of Pathology, Nihon University School of Medicine, Tokyo, Japan

**Keywords:** Fever of unknown origin, Malignant giant cell tumor, Malignant transformation, Naproxen, Neoplastic fever

## Abstract

**Background:**

Malignant transformation of giant cell tumors of bones, that is, secondary malignant giant cell tumor of bone, is rare. The most common symptoms are local pain and swelling. There are no prior reports of giant cell tumor of bone with fever of unknown origin at the onset. Here we present a case of a secondary malignant giant cell tumor of bone due to malignant transformation 40 years after surgery without radiation therapy, presenting as fever of unknown origin.

**Case presentation:**

A 75-year-old Asian man presented with a 3-week history of continuous pyrexia and left knee pain and swelling. He had been diagnosed at age 35 years with a giant cell tumor of bone of his left distal femur and underwent bone curettage and avascular fibula grafting at that time. Postoperative radiation therapy was not performed. He remained recurrence-free for 40 years after surgery. At age 75, histopathological findings suggested a secondary malignant giant cell tumor of bone. The tumor specimen expressed tumor necrosis factor-α. Neoplastic fever was suspected, and a naproxen test was conducted. His pyrexia showed immediate resolution. Surgery was performed under a diagnosis of a secondary malignant giant cell tumor of bone with neoplastic fever. His pyrexia and inflammatory activities diminished postoperatively.

**Conclusions:**

This is the first reported case, to the best of our knowledge, of the detection of a secondary malignant giant cell tumor of bone based on fever of unknown origin after long-term (40 years) follow-up. After curettage and bone grafting, giant cell tumor of bone may transform to malignancies within a few years or even decades after surgery. Therefore, meticulous follow-up is essential. The fever might be attributable to the tumor releasing inflammatory cytokines. Not only pain and swelling but also continuous pyrexia may suggest the diagnosis of a secondary malignant giant cell tumor of bone.

## Background

Giant cell tumors of bones (GCTBs) are generally benign, but there are rare cases showing malignant transformation during long-term follow-up. Malignant GCTBs are classified into two types: primary and secondary tumors. Primary malignant GCTB consists of the benign form with a malignant component which is simultaneously present and gradually expands. Secondary malignant GCTBs result from malignant transformation after various periods following initial treatment for a benign GCTB, and the reported incidences are very low (0.5 to 5 %) [1, 2]. The interval until malignant transformation ranges from 10 to 41 years, that is, malignancies can develop even during very long-term follow-up [3, 4]. The most common symptoms of secondary malignant GCTBs are local pain and swelling [5]. However, to the best of our knowledge, there are no reports of secondary malignant GCTB presenting as fever of unknown origin (FUO). Here we describe a patient with FUO who showed malignant transformation 40 years after surgery for a GCTB. Moreover, continuous pyrexia excluded infection and allergic reactions from the possible differential diagnoses and distinguished malignant GCTB from the benign form of this tumor.

## Case presentation

A 75-year-old Asian man presented with a 3-week history of continuous pyrexia and left knee pain and swelling. He had been diagnosed with a GCTB of his left distal femur at age 35 years and was treated with bone curettage and avascular fibula grafting at that time. Postoperative radiation therapy was not performed. He remained recurrence-free for 40 years after surgery. He also had a history of fibrous dysplasia of the craniofacial bones at 35 years of age. He neither smoked cigarettes nor drank alcohol. At the initial consultation for FUO, his temperature was 38.3 °C, and a slight heat sensation and swelling were noted around his left knee. The range of motion of his left knee was restricted to 5 to 70 degrees. A patellar tap test, for fluid in the knee, was positive. Articular puncture was performed and the fluid obtained was cultured; however, no bacteria were identified. His leukocyte count was 5600/μL (4000 to 8000/μL) and C-reactive protein (CRP) was 17.8 mg/dL (<0.2 mg/dL), suggesting increased inflammatory activities. There were no other abnormalities.

**Fig. 1:**
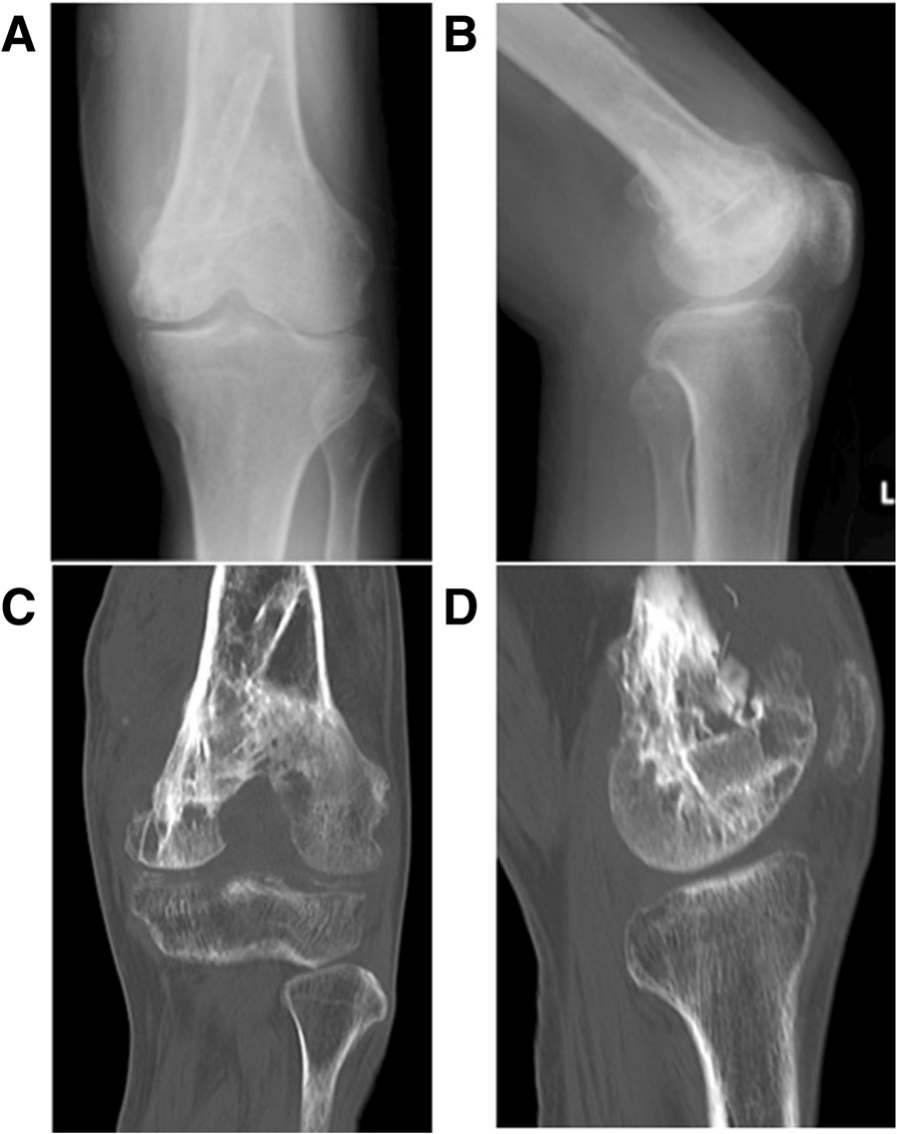
Plain X-ray findings and computed tomography findings. Plain X-ray (**a**, **b**) and computed tomography (**c**, **d**) show bone grafts, including a fibula graft performed at the time of initial surgery involving transplantation of the femoral metaphysis to the epiphyseal area. Neither bone translucency nor destruction is apparent on these images

**Fig. 2:**
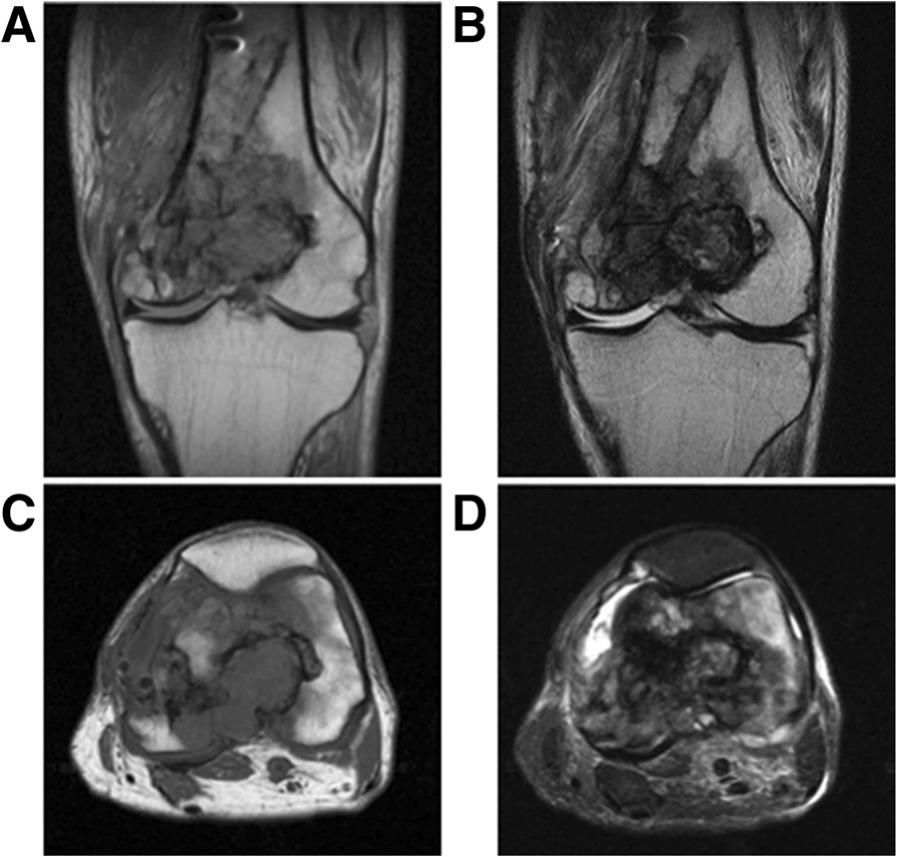
Magnetic resonance imaging. Coronal section: **a** T1-weighted image, **b** T2-weighted image. Transverse section: **c** T1-weighted image, **d** short T1 inversion recovery. Fluid retention can be seen in the medial femur and intra-articular area. T1-weighted images of the intra-osseous area show a low to isosignal intensity, and T2-weighted images showed an isosignal to high signal intensity. There are no masses in the extra-osseous area

**Fig. 3:**
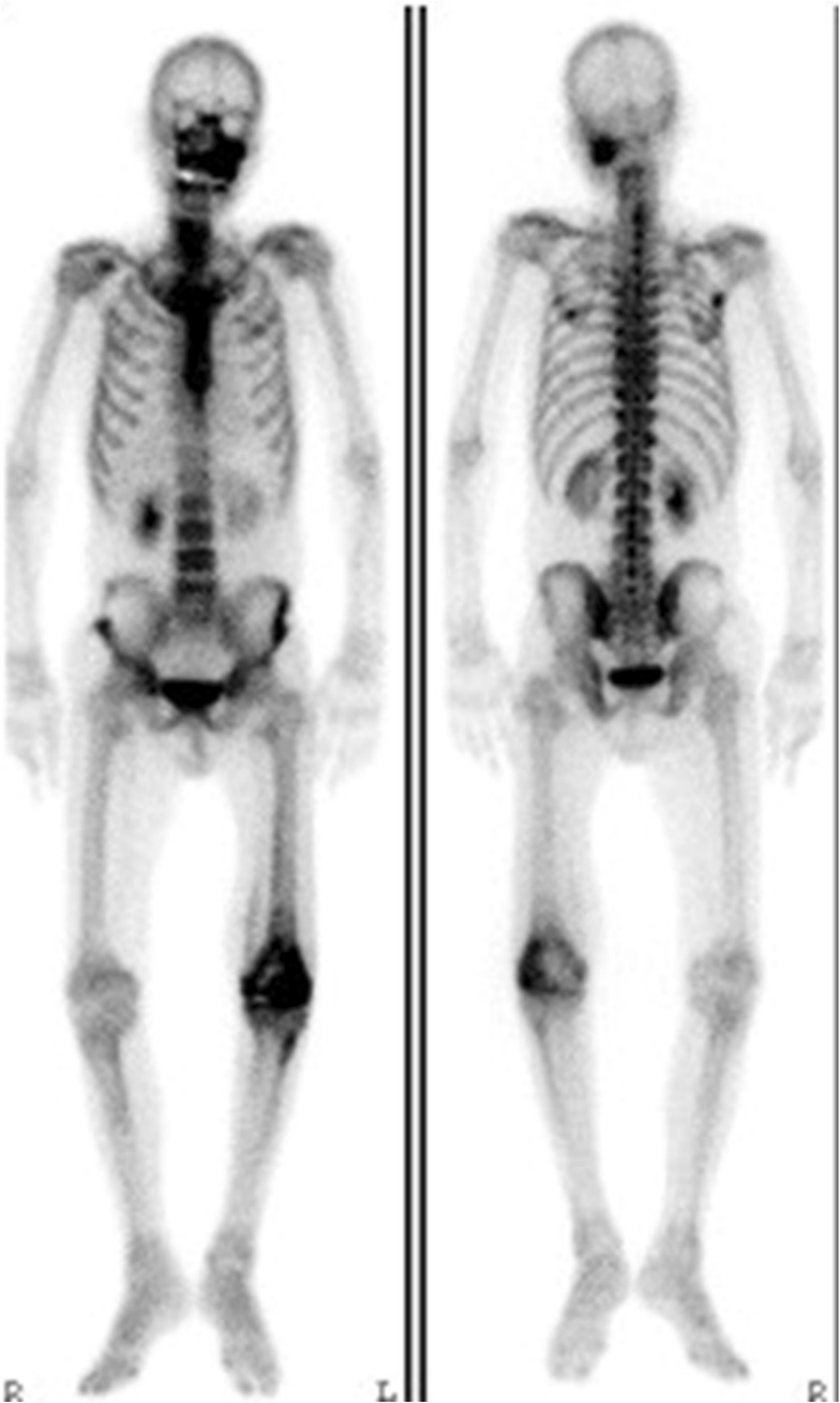
Bone scintigraphy. There is abnormal accumulation in the left distal femur. The abnormal accumulation in the craniofacial bones was attributed to fibrous dysplasia

**Fig. 4:**
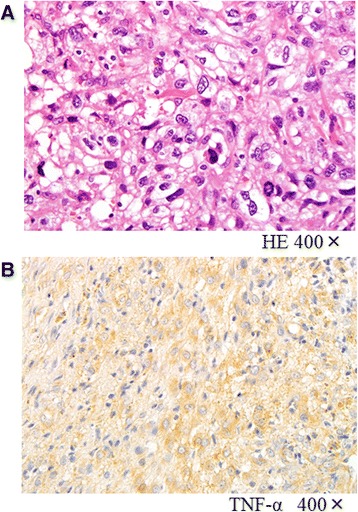
Histopathological findings. **a** Hematoxylin and eosin staining (×400): neither tumor osteoid formation nor residual areas of giant cell tumors of bone are present. There is dense proliferation of tumor cells with atypia/nuclear division. Undifferentiated pleomorphic sarcoma transformation was diagnosed. **b** Tumor necrosis factor-α staining (×400): the tumor specimen expressed tumor necrosis factor-α. *HE* hematoxylin and eosin, *TNF-α* tumor necrosis factor-α.

A plain X-ray and computed tomography (CT) showed bone grafts, including a fibula graft from the femoral metaphysis to the epiphyseal area that had been performed at the time of the initial surgery 40 years earlier. Neither bone translucency nor destruction was detected (Fig. 1). Magnetic resonance imaging (MRI) revealed fluid retention in his medial femur and intra-articular area. T1-weighted images of the intra-osseous area showed a low to isosignal intensity, and T2-weighted images showed an isosignal to high signal intensity. There were no masses in the extra-osseous area (Fig. 2). Bone scintigraphy revealed an abnormal accumulation in his left distal femur (Fig. 3). Although his bacterial culture revealed no infectious organisms, based on findings including local symptoms and the inflammatory activities, surgical debridement in addition to antibiotic treatment was performed under clinical suspicion of chronic osteomyelitis of the distal femur. However, the pyrexia persisted. On histopathological examination, neither tumor osteoid formation nor residual areas of GCTB were identified, but dense proliferation of tumor cells with atypia/nuclear division was indicative of malignant transformation to undifferentiated pleomorphic sarcoma. Thus, rather than chronic osteomyelitis, a secondary malignant GCTB was diagnosed (Fig. 4a). Furthermore, the tumor specimen expressed tumor necrosis factor-α (TNF-α; Fig. 4b). Neoplastic fever was suspected, and a naproxen test was thus conducted. His pyrexia subsided within 24 hours of administration. There were no metastases except in his left distal femur. Under a diagnosis of a secondary malignant GCTB with neoplastic fever, his left femur was amputated. Unfortunately, a limb salvage procedure was not feasible due to widespread dissemination of malignant cells caused by the previous surgical debridement. There was no fever postoperatively, and inflammatory activities diminished markedly. To date, his course has been favorable.

## Discussion

GCTBs are benign bone tumors consisting of two cellular components: interstitial tumor cells and a large number of multinuclear giant cells. These tumors most frequently develop in individuals of 20 to 39 years of age. Common tumor sites are the distal femur and proximal tibia. Local recurrence rates range from 10 to 25 %. The interval until local recurrence is reportedly 6 months or less in 25 % of these patients and 2 years or less in 97 % of these patients [6]. Therefore, relapse after 5 years or more of follow-up is extremely rare [1, 2].

Secondary malignant GCTBs, with incidences ranging from 0.5 to 5 %, represent transformation from the benign to the malignant form of GCTB after surgical treatment [1, 2]. The reported incidences include patients who underwent surgical treatment both with and without radiation therapy. The development of malignancy a long time after radiation therapy has been recognized. Incidences in patients undergoing surgery alone without radiation therapy are only 0.2 to 2 % [3, 7, 8]. According to previous reports, transformation to a secondary malignant GCTB can occur without radiation therapy 10 to 41 years after treatment [3, 4]. GCTBs may also recur during long-term follow-up. Hence, it is necessary to discriminate between benign and malignant GCTB in some cases [1, 2]. As previously reported, the most common primary symptoms of the malignant form are pain and swelling [5]. However, to the best of our knowledge, there are no prior reports describing a patient such as ours with continuous pyrexia as a primary symptom. The prognosis of patients with secondary malignant GCTB is poor [3]. There are no characteristic symptoms or imaging findings, which makes early detection difficult [3]. Therefore, continuous pyrexia not due to either infection or an allergic reaction may facilitate distinguishing malignant GCTB from the benign form.

Although the mechanism of malignant transformation remains to be clarified, bone infarction is known to be involved in the development of sarcomas, such as osteosarcoma, malignant fibrous histiocytoma, and fibrosarcoma [9, 10]. Furthermore, a previous study documented malignant transformation at the site of bone grafting [11]. The grafted bone cells may have died, resulting in malignant transformation through repair and growth-related changes. Our patient had undergone curettage and fibula grafting, such that malignant cells may have aggregated at the site of bone grafting. Malignancy may thereby have developed 40 years after initial treatment.

FUO is defined as “fever persisting for 3 weeks or more and reaching 38.3°C or higher at least three times, based on which a definitive diagnosis cannot be made despite admission/detailed examination for 1 week”. In 60 % of such patients, FUO is related to infection. However, neoplastic fever accounts for 27 % of those with non-infectious fever; this percentage is relatively high [12]. Diagnostic criteria for neoplastic fever are presented in Table 1. On a naproxen test, naproxen is administered, and reactions are regarded as positive if pyrexia diminishes 24 hours after administration. The sensitivity and specificity of this test are reportedly 92 and 100 %, respectively. The interval from naproxen administration until antipyretic activity is shorter than that from diclofenac or indomethacin administration [13]. Antipyretic activity was reportedly achieved in 50 % of patients treated with steroids, but 90 % of patients treated with naproxen. Our patient showed a positive reaction on the naproxen test, meeting the diagnostic criteria for neoplastic fever.

**Table 1:** Diagnostic criteria for neoplastic fever

Temperature over 37.8 °C at least once each day
Duration of fever over 2 weeks
Lack of evidence of infection (physical examination, laboratory examinations, imaging studies)
Absence of allergic mechanisms (drug allergy, transfusion reaction, and radiation or chemotherapeutic drug reaction)
Lack of response of fever to an empiric, adequate antibiotic therapy for at least 7 days
Prompt complete lysis of fever by the naproxen test with sustained normal temperature while receiving naproxen

**Fig. 5:**
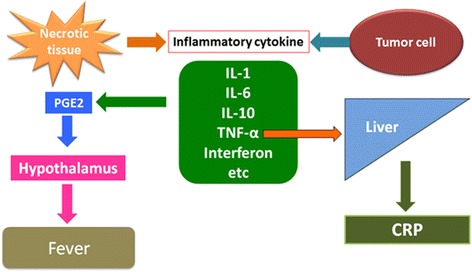
Mechanism of neoplastic fever. Inflammatory cytokines are produced by necrotic tissue or tumor cells, and these factors act on the hypothalamus through prostaglandin E2 induction, thereby by causing fever by raising the set body temperature. Furthermore, tumor necrosis factor-α acts on the liver, producing C-reactive protein and thus increasing the serum C-reactive protein level. *CRP* C-reactive protein, *IL-1* interleukin-1, *IL-6* interleukin-6, *IL-10* interleukin-10, *PGE2* prostaglandin E2, *TNF-α* tumor necrosis factor-α

Although the pathogenesis of neoplastic fever remains to be clarified, inflammatory cytokines, such as interleukin-1 (IL-1), IL-6, TNF-α, and interferon, are reportedly involved. Inflammatory cytokines are produced by necrotic tissue or tumor cells, and these factors act on the hypothalamus through prostaglandin E2 (PGE2) induction, inducing fever by raising the set body temperature. Furthermore, TNF-α acts on the liver, producing CRP and thereby increasing the CRP level (Fig. 5). The incidence of neoplastic fever in patients with sarcoma, among malignant neoplasms, is reportedly 3.5 % [14]. A prior patient in our care, who had initially complained of FUO, was found to have a malignant fibrous histiocytoma. Expression of inflammatory cytokines, such as TNF-α, was detected at the tumor site [15]. TNF-α was also expressed at the tumor site in the present case (Fig. 5). After amputation, our patient’s inflammatory response diminished. Therefore, the tumor may have produced inflammatory cytokines.

## Conclusions

This is the first report to describe a secondary malignant GCTB detected based on FUO after very long-term (40 years) follow-up. GCTBs treated by curettage and bone grafting may become malignant over several decades following surgery. Therefore, meticulous follow-up is essential. The origin of the fever might have been the release of inflammatory cytokines from the tumor itself. Not only pain and swelling but also continuous pyrexia may facilitate the diagnosis of malignant transformation of GCTBs.

## Consent

Written informed consent was obtained from the patient for publication of this case report and accompanying images. A copy of the written consent is available for review by the Editor-in-Chief of this journal.

## Abbreviations

CRP: C-reactive protein
CT: Computed tomography
FUO: Fever of unknown origin
GCTBs: Giant cell tumors of bones
IL-1: Interleukin-1
MRI: Magnetic resonance imaging
PGE2: Prostaglandin E2
TNF-α: Tumor necrosis factor-α
